# Evolutionary conservation hotspots: key areas for threatened Neotropical glassfrogs under climate change scenarios

**DOI:** 10.7717/peerj.21165

**Published:** 2026-04-28

**Authors:** Mateo A. Vega-Yánez, Julián A. Velasco, Carl R. Hutter, Daniela Franco-Mena, Luis Amador, Juan M. Guayasamin

**Affiliations:** 1 Instituto Nacional de Biodiversidad, Quito, Ecuador; 2 Colegio de Ciencias Biológicas y Ambientales COCIBA, Maestría en Ecología Tropical y Conservación, Universidad San Francisco de Quito, Quito, Ecuador; 3 Instituto de Ciencias de la Atmósfera y Cambio Climático, Universidad Nacional Autónoma de México, Mexico City, Mexico; 4 Department of Biological Sciences and Museum of Natural Science, Louisiana State University, Baton Rouge, LA, United States of America; 5 Virus and Prion Research Unit, National Animal Disease Center, Agricultural Research Service, United States Department of Agriculture, Ames, IA, United States of America; 6 Laboratorio de Biología Evolutiva, Colegio de Ciencias Biológicas y Ambientales COCIBA, Global Research & Solutions Center, Universidad San Francisco de Quito, Quito, Ecuador; 7 Museum of Southwestern Biology and Department of Biology, University of New Mexico, Albuquerque, NM, United States of America

**Keywords:** Climate change, Biodiversity, Conservation, Phylogenetics, Tropical Andes

## Abstract

Anthropogenic climate change is one of the main threats to global biodiversity, with amphibians being among the most vulnerable vertebrate groups. In this context, the IUCN currently lists 69 species of Neotropical glassfrogs as threatened. However, our knowledge of how their taxonomic and phylogenetic diversity will be distributed in future climate change scenarios remains limited. In this study, we projected the future distribution of threatened species to estimate changes in taxonomic and phylogenetic diversity across geography under two climate scenarios (SSP2-4.5 and SSP3-7.0). We also identified priority areas for conservation based on phylogenetic diversity and the Evolutionary and Global Distinctiveness Index. Our results suggest that the Andes and Amazon Basin will experience the most drastic climatic changes, with at least six species projected to experience complete loss of climatic suitability across all assessed scenarios, consequently facing a high risk of extinction. Additionally, most species exhibit a tendency to shift towards higher elevations, accompanied by a significant reduction in their geographic range. On average, these changes could result in a loss of approximately 30% of their phylogenetic diversity. The northwest Andean montane forests of Ecuador and Colombia are identified as key refuges for future taxonomic and phylogenetic diversity of glassfrogs. However, less than 36% of their projected range overlaps with protected areas, highlighting the immediate need for conservation action.

## Introduction

Anthropogenic climate change has contributed to the decline of numerous amphibian species by reducing climatically suitable habitats (Carey & Alexander, 2003; Luedtke et al., 2023). The effects of climate change are expected to have a higher impact in neotropical regions (Velasco et al., 2021). For example, montane species will experience drastic shifts in their distribution, being pushed upward to follow their climatic niches as temperatures rise (Menéndez-Guerrero, Davies & Green, 2020). There is also a potential risk of losing suitable areas for species adapted to mountain environments (Forero-Medina, Joppa & Pimm, 2011; Cordier et al., 2020; De Meyer, Ortega-Andrade & Moulatlet, 2022), while lowland species may progressively shift toward higher elevations or latitudes. In addition, some species may face particular challenges under climate change due to climatic niche conservatism, which refers to the tendency of lineages to retain ancestral climatic tolerances over evolutionary time (Hutter, Guayasamin & Wiens, 2013). Because this limits their ability to adapt rapidly to novel climatic conditions, these species are more likely to respond by tracking suitable environments through geographic range shifts (Wiens & Graham, 2005). Consequently, they may need to move considerable distances and could face a higher risk of extinction when dispersal ability is low (Bonetti & Wiens, 2014; Antão et al., 2020). This challenge is particularly relevant for glassfrogs (Centrolenidae family), a group comprising approximately 168 species distributed across Central and South America (Guayasamin et al., 2020; Frost, 2026). Their diversification has been strongly influenced by the uplift of the Andes, the heterogeneity of the landscape, and evolutionary processes linked to niche conservatism, which have promoted allopatric speciation and led to high species richness, especially at intermediate elevations (Hutter, Guayasamin & Wiens, 2013; Guayasamin et al., 2020). Moreover, patterns of phylogenetic diversity and endemism underscore the ecological importance of the northern Andes and the humid forests of the Chocó-Darién region for this lineage (Mendoza & Arita, 2014).

Despite this diversity, glassfrogs face severe conservation challenges. Around 44% of species are currently considered globally threatened (IUCN, 2026). Specifically, 11 species are listed as Critically Endangered (CR), 39 as Endangered (EN), and 19 as Vulnerable (VU), mainly due to habitat loss, emerging diseases, and the impacts of climate change (Wake, 2007; Delia Basanta, Velasco & González-Salazar, 2023; Luedtke et al., 2023). In particular, the potential effects of climate change on the future distribution of glassfrogs remain poorly explored. As an Andean clade (Hutter, Guayasamin & Wiens, 2013; Castroviejo-Fisher et al., 2014), these species may face a substantial risk of losing suitable habitat under changing climatic conditions (Forero-Medina, Joppa & Pimm, 2011; Ortega-Andrade, Rojas-Soto & Paucar, 2013). Understanding these dynamics is crucial to evaluate how climate change could affect the distribution of threatened lineages, particularly those exhibiting strong niche conservatism. Furthermore, it is essential to identify which areas in Neotropics harbor the highest taxonomic and phylogenetic diversity of threatened glassfrogs. In this study, priority areas are identified using spatial overlap of species distribution models and metrics of phylogenetic diversity under current and future climate scenarios. However, spatial prioritization under climate change remains subject to uncertainties related to model projections, dispersal assumptions, and data limitations.

## Materials and Methods

### Data compilation

Based on the IUCN Red List for the Centrolenidae family, we compiled records of the presence of the 69 threatened species from biodiversity databases, including the Global Biodiversity Information Facility (GBIF) and other similar platforms, and from scientific museum collections (Instituto Nacional de Biodiversidad, Museo de Historia Natural La Salle, Instituto de Ciencias Naturales) and literature. Complete details are presented in Supplementary S1. With this data, taxonomic validation was carried out with the aim of updating the nomenclature of genera and species, considering that some taxonomies have been revised, reclassified, or synonymized in recent years. In addition, geographic coordinates were validated in ArcGIS Pro and duplicate records were removed to ensure a robust database for subsequent analyses. For each species, the M area or accessibility area (Soberón & Peterson, 2005) was defined using a 500 km buffer.

### Climatic variables

Bioclimatic variables were obtained from the WorldClim version 2.1 platform (Fick & Hijmans, 2017) at a spatial resolution of 2.5 min. For the future climate projections, we downloaded data from the CMIP6 models (Eyring et al., 2016) at the same spatial resolution as the current variables, for the time horizon 2061–2080 under two Shared Socio-economic Pathways (SSP2-4.5 and SSP3-7.0) (Eyring et al., 2016). SSP2-4.5 represents an intermediate scenario, in which climate policies implemented are moderate. In this context, the medium use of fossil fuels is maintained, and sustainable development advances partially. Whereas SSP3-7.0 corresponds to a high impact scenario in which weak or minimal climate change policies are observed. This scenario is marked by intensive use of fossil fuels and limited progress in terms of sustainable development (Riahi et al., 2017).

We selected two Global Climate Models (GCMs); CMCC-ESM2 and GISS-E2-1-G at random. The first model, CMCC-ESM2 has a higher equilibrium climate sensitivity (ECS), which leads to a more perceptible temperature increase compared to GISS-E2-1-G. This higher sensitivity induces more intense changes in atmospheric circulation and moisture distribution (Lovato et al., 2022). It is also worth noting that CMCC-ESM2 includes a more detailed representation of terrestrial biogeochemical cycles, which affects evapotranspiration and albedo, reducing the availability of moisture in the atmosphere and decreasing precipitation in some areas. While the GISS-E2-1-G model simulates a stronger indirect effect of aerosols on clouds, which may contribute to increased precipitation (Lovato et al., 2022; Nazarenko et al., 2022). To explore changes in climatic conditions relative to the present, we used the raster package in R (Hijmans, 2024) to perform map algebra and calculate differences in temperature and precipitation for each GCM under each SSP scenario.

### Species distribution modeling

According to area M, a series of models were generated in R software (R Core Team, 2024). Species with fewer than five occurrence records were not modeled and were instead analyzed only under the current potential distribution. For these species, occurrence records were converted into pixels at a spatial resolution of 2.5 min. While for the species modeled, we adopted a model ensemble approach due to the significant uncertainty in the selection of model algorithms in relation to their transferability to climate change scenarios (Thuiller et al., 2019; Castelblanco-Martínez et al., 2021). First, with the R package ecospat (Broennimann, Di Cola & Guisan, 2024) we generated 1,000 random pseudo-absences for each species, ensuring a minimum distance of ∼4.5 km from confirmed occurrence records. We then used the sdm package (Naimi & Araújo, 2016) to run three algorithms: Boosted Regression Trees (BRT), Random Forest (RF) and Support Vector Machine (SVM) with subsampling replication where 30% of the data were used for validation, and the remaining 70% for training, generating a total of 10 replicates for each algorithm (Engler et al., 2013). Finally, for each individual model we evaluated the predictive accuracy using the TSS and the omission rate (Allouche, Tsoar & Kadmon, 2006). We also generated an ensemble model weighting for those models that maximize TSS values to obtain the model ensemble current for each glassfrog species. For the future climate projections, we performed a series of model transfers for the two GCMs (CMCC-ESM2 and GISS-E2-1-G) in each SSP (245 and 370). The code used for data analyses in this study is publicly available in a GitHub repository (https://github.com/mateovegayanez/-Key-areas-for-threatened-Neotropical-glassfrogs.git). Using consensus assemblage models for each species under current and future distributions, binary maps were generated with the R package ecospat, applying a threshold of 0.9 based on species occurrences for the analysis.

### Phylogenetic analyses

Mitochondrial DNA sequences of glassfrogs reported in previous studies (Guayasamin et al., 2020), along with those of the outgroups, were obtained from GenBank (http://www.ncbi.nlm.nih.gov/genbank/) (Supplementary S2). DNA sequence data represents coverage of 80.7% of the Centrolenidae family. Taxon sampling comprised 134 described glassfrog species, eleven putative new species, and three outgroup taxa. The dataset contains complete or partial sequences of three genes representing 2,569 bp of data (mitochondrial: 12S rRNA, 16S rRNA, and ND1). The alignments of the three mitochondrial genes were previously reviewed and edited in Aliview (Larsson, 2014) and then concatenated in the AMAS program (Borowiec, 2016) for subsequent phylogenetic analyses.

Maximum Likelihood analysis was performed in IQ-TREE version 2.2.0 (Minh et al., 2020) under the GTR model and default values. Node support was evaluated as 1,000 ultrafast bootstrap replicates (Hoang et al., 2018). Bayesian Inference analysis was obtained with BEAST 2 (Bouckaert et al., 2019) implemented with a run of 5.0 × 10^7^ generations sampled every 1,000 generations; topological convergence to a stable zone was analyzed in Tracer (Rambaut et al., 2018). For the time-calibrated ultrametric tree, the temporal calibration scheme described by Castroviejo-Fisher et al. (2014) for the most recent common ancestor of *Centrolene* was used. The maximum clade credibility tree (MCC) was estimated with TreeAnnotator v2 (distributed with BEAST 2) with the trees sampled after discarding 25% as burn-in. Species with their identification unconfirmed (*e.g.*, cf. or aff. species) were removed with the drop.tip function of the ape package in R (Paradis & Schliep, 2019). The Bayesian and Maximum Likelihood tree was visualized using FigTree v1.4.3 (http://tree.bio.ed.ac.uk/software/figtree/).

### Geographical patterns of taxonomic and phylogenetic diversity

Binary maps were generated for each species and subsequently stacked to produce a presence–absence matrix (PAM). This PAM was used to estimate taxonomic diversity (TD) and as the basis for subsequent analyses. Species that could not be modeled due to the small number of records were excluded from future distribution analyses.

Using the phylogeny that included threatened species, we calculated phylogenetic diversity (PD) as the sum of the branch lengths of all species present in the PAM (Faith, 1992). To examine the relationship between PD and TD, we applied a Locally Estimated Scatterplot Smoothing (LOESS). In this framework, positive residuals indicate few recent speciation events and/or high dispersal rates, whereas negative residuals suggest numerous recent speciation events and/or low dispersal rates (Ochoa-Ochoa et al., 2020). Finally, residual values were rasterized to generate maps for each GCMs at a spatial resolution of 0.5 degrees latitude.

### Evolutionarily Distinct and Globally Endangered

The Evolutionary Distinctiveness (ED) index was calculated for each threatened glassfrog species using the picante package in R (Kembel et al., 2010), based on its phylogeny. This index distributes branch lengths of the phylogenetic tree equally among descendant branches, applying proportional and fair splits (Isaac et al., 2007; Redding, Mazel & Mooers, 2014). The aim is to measure the degree of isolation of each species within the phylogeny, thus reflecting its evolutionary uniqueness (Molina-Venegas, 2021). The Globally Endangered (GE) for each species was assigned according to the probability of extinction for 100 years, following the criteria established by Mooers, Faith & Maddison (2008). With these values, the Evolutionarily Distinct and Globally Endangered (EDGE) was calculated for each species by ln (1 + ED) + GE × ln (2) (Gumbs et al., 2023). With the EDGE values, an analysis of the distribution of continuous characters along the phylogenetic tree of the threatened species was performed using the phylotools package in R (Zhang, 2017). The values were rasterized in the grid cells generated from the species distribution model for the current, using a resolution of 0.5 degrees latitude.

## Results

### Temperature and precipitation differences between GCMs and the present

#### Temperature

**Figure 1:**
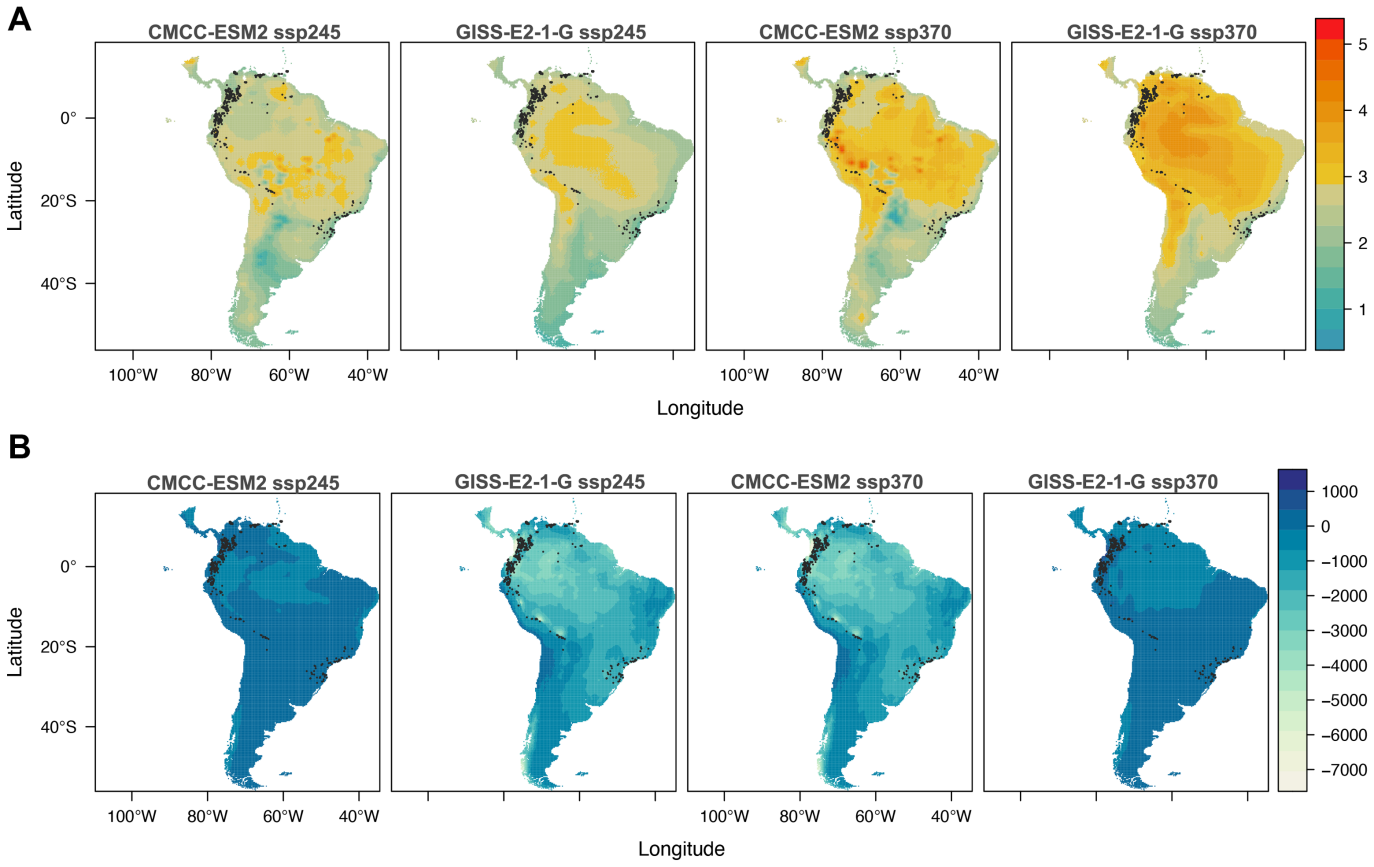
Difference between Global Climate Models (GCMs) and Shared Socio-economic Pathways (SSP) by 2061–2080, with respect to current climate conditions. (A) Temperature and (B) precipitation. Dark gray dots show records of occurrences of threatened glassfrogs.

Under both SSP2-4.5 and SSP3-7.0 scenarios, climate models consistently project substantial warming across South America, with the most pronounced increases concentrated in the central Andes, the Guiana Shield, and extensive areas of the Amazon basin (Fig. 1A). Under SSP2-4.5, temperatures are projected to rise by approximately 3–4 °C, increasing to about 4–5 °C under SSP3-7.0. Threatened glassfrog species, particularly those classified as Critically Endangered (CR) and Endangered (EN), are expected to experience the highest temperature increases.

#### Precipitation

Precipitation projections under both SSP2-4.5 and SSP3-7.0 show substantial model variability but consistently indicate drying trends across the Amazon and northeastern South America, with reductions reaching several hundred millimeters and intensifying under the high-emissions scenario. For glassfrogs, projected precipitation declines mainly affect Endangered (EN) species, whereas projected increases may impact both Critically Endangered (CR) and (EN) taxa (Fig. 1B; Table 1).

**Table 1:** Differences between average temperature and precipitation values by threat category for each global climate models (GCMs) and Shared Socio-economic Pathways (SSPs).

	**SSP2-4.5**		**SSP3-7.0**		**SSP2-4.5**		**SSP3-7.0**	
	CMCC-ESM2	GISS-E2-1-G	CMCC-ESM2	GISS-E2-1-G	CMCC-ESM2	GISS-E2-1-G	CMCC-ESM2	GISS-E2-1-G
	Temperature ° C				Precipitation mm			
Critically Endangered	2.497	2.433	2.737	2.960	−78.010	172.833	−145.446	208.235
Endangered	2.509	2.437	2.763	2.986	−123.080	187.849	−195.402	238.279
Vulnerable	2.232	2.274	2.417	2.759	−21.464	50.456	−76.525	15.257

### Phylogenetics

**Figure 2:**
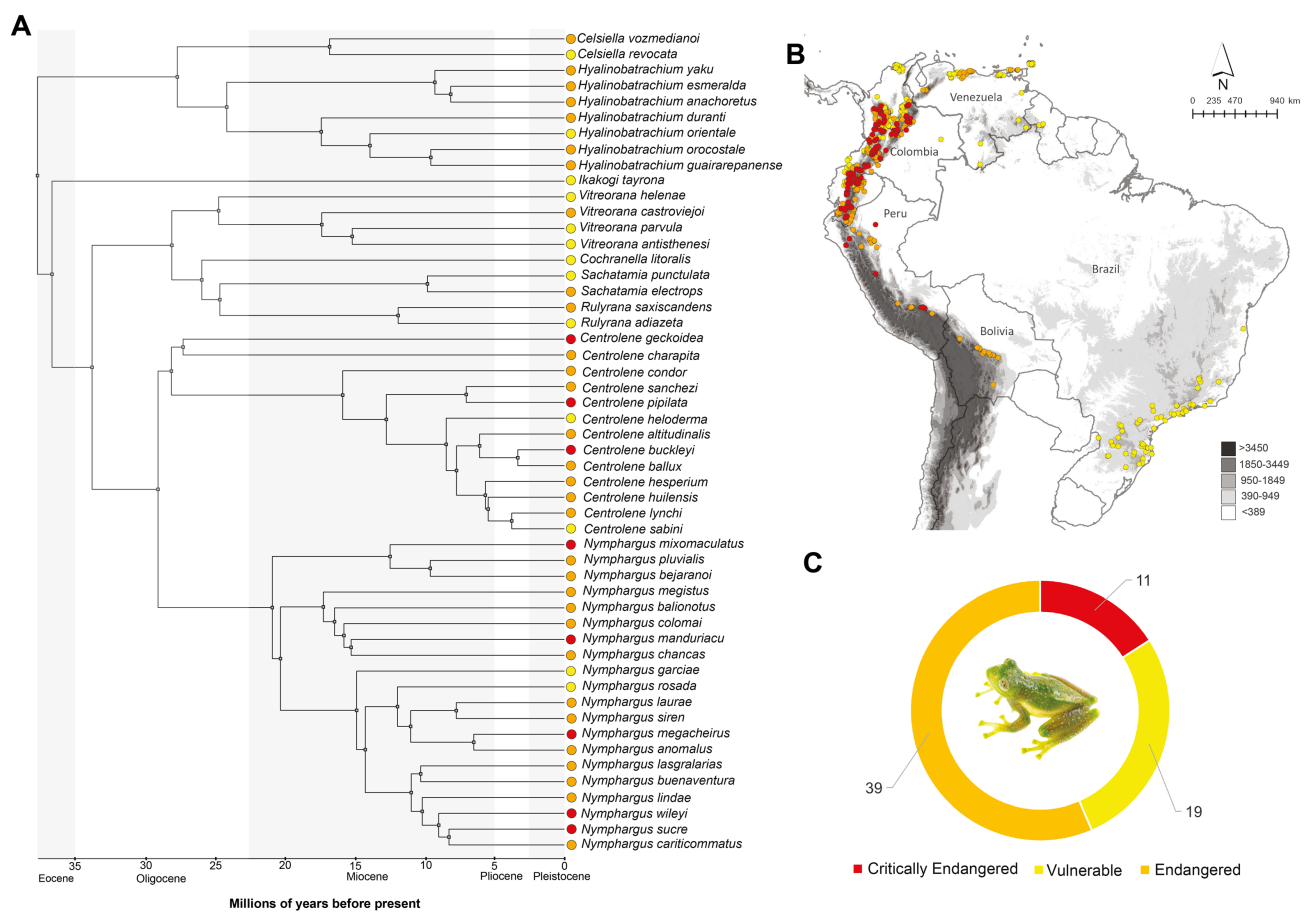
(A) Phylogeny and estimated divergence times of threatened glassfrogs (Vulnerable, Endangered and Critically Endangered). (B) Map of threatened species occurrence records (C) Number of glassfrog species by threat IUCN category.

Of 69 threatened species in total, 52 had genetic sequences available. Phylogenetic relationships of threatened glassfrog species show that the highest diversification occurred during the Miocene (∼23 Ma) (Fig. 2A). The ancient species *is Ikakogi tayrona*, which occurred in the Eocene, approximately 36.7 Ma. Currently, this species is distributed in northern Colombia, specifically in the Sierra Nevada, and is classified as Vulnerable (VU) (Fig. 2B). The subfamily Hyalinobatrachinae, which includes the genera *Celsiella* and *Hyalinobatrachium*, is composed of species currently classified as Endangered (EN) and Vulnerable (VU). This subfamily diverged approximately 27.7 Ma. The other subfamily, Centroleninae, diverged approximately 33.8 Ma and comprised all other genera of threatened species (*Centrolene*, *Cochranella*, *Nymphargus*, *Rulyrana*, *Sachatamia* and *Vitreorana*), represented across all three IUCN threat categories (CR, EN, VU). Within this subfamily, the *Centrolene* genus clade includes four species (*Centrolene buckleyi*, *Centrolene ballux*, *Centrolene lynchi*, and *Centrolene sabini*) that represent the most recent lineages, with a diversification that originated in the Pliocene, approximately 5 Ma. These species are classified in the three threat IUCN categories (Fig. 2C).

### Occurrence record data

A total of 986 validated occurrence records were obtained for the distribution models of threatened glassfrog species in the Neotropics, classified as follows: 430 records corresponding to Vulnerable species, 352 to Endangered species and 204 to Critically Endangered species. North of the Andes, in Ecuador and Colombia, there is the highest concentration of species classified as Critically Endangered (CR) (Figs. 2B–2C). However, the majority of threatened glassfrog species (39) are in the Endangered (EN) category, widely distributed throughout the Andes, from the south (Bolivia and Peru) to the north (Ecuador, Colombia and Venezuela). Records of occurrence in the Vulnerable (VU) category (19 species) are similarly distributed throughout the Andes. Records of occurrence in Brazil correspond to only one species (*Vitreorana parvula*).

### Species distribution models

#### Taxonomic diversity

Fifty-five percent of the threatened glassfrog species (38 species) were modeled. Subsequently, these models were projected for the two climate change scenarios (SSP2-4.5 and SSP3-7.0) and for the two general circulation models (CMCC-ESM2 and GISS-E2-1-G).

#### Current potential scenario

**Figure 3:**
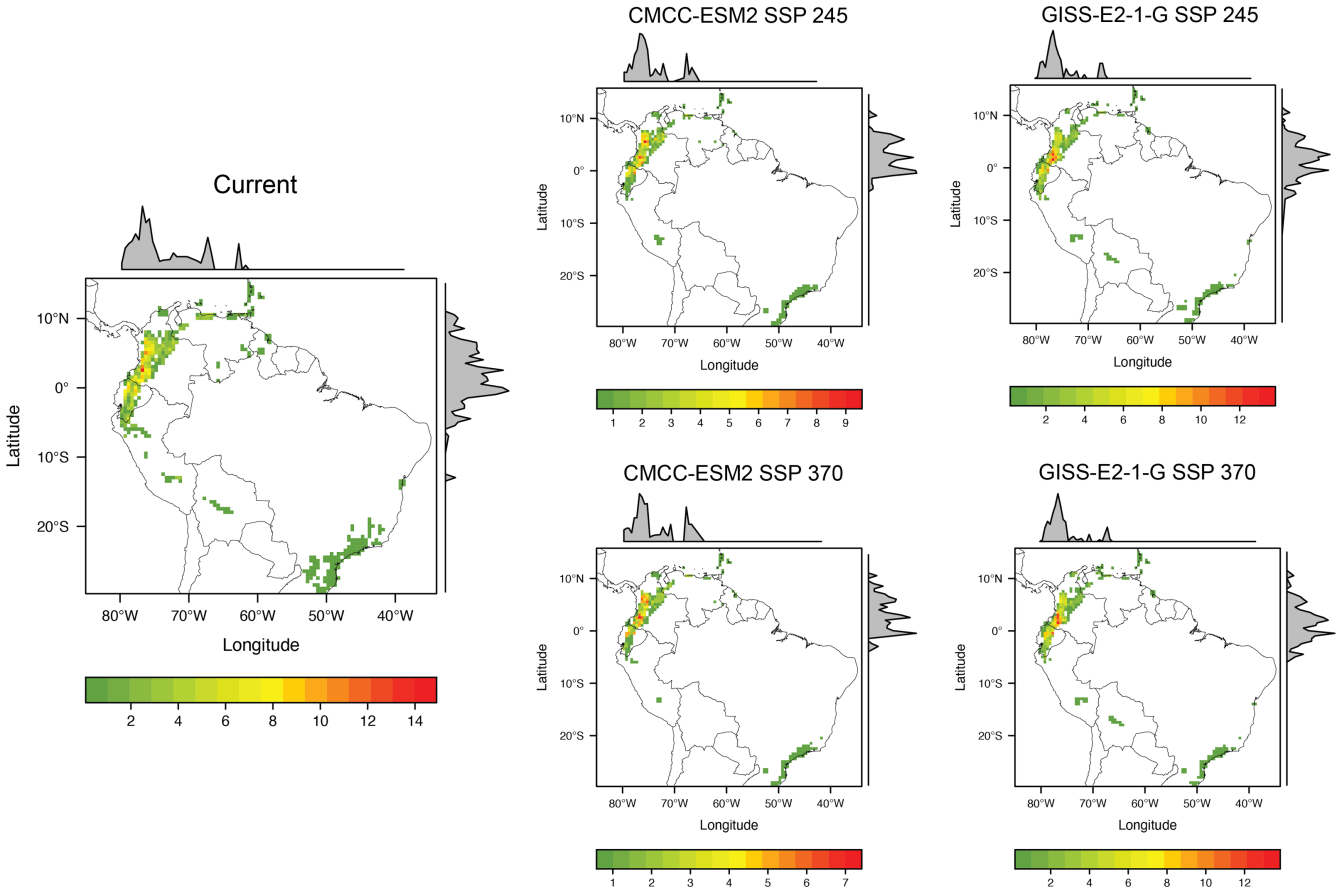
Current and future distribution pattern of taxonomic diversity (TD) of threatened Neotropical glassfrogs species.

**Figure 4:**
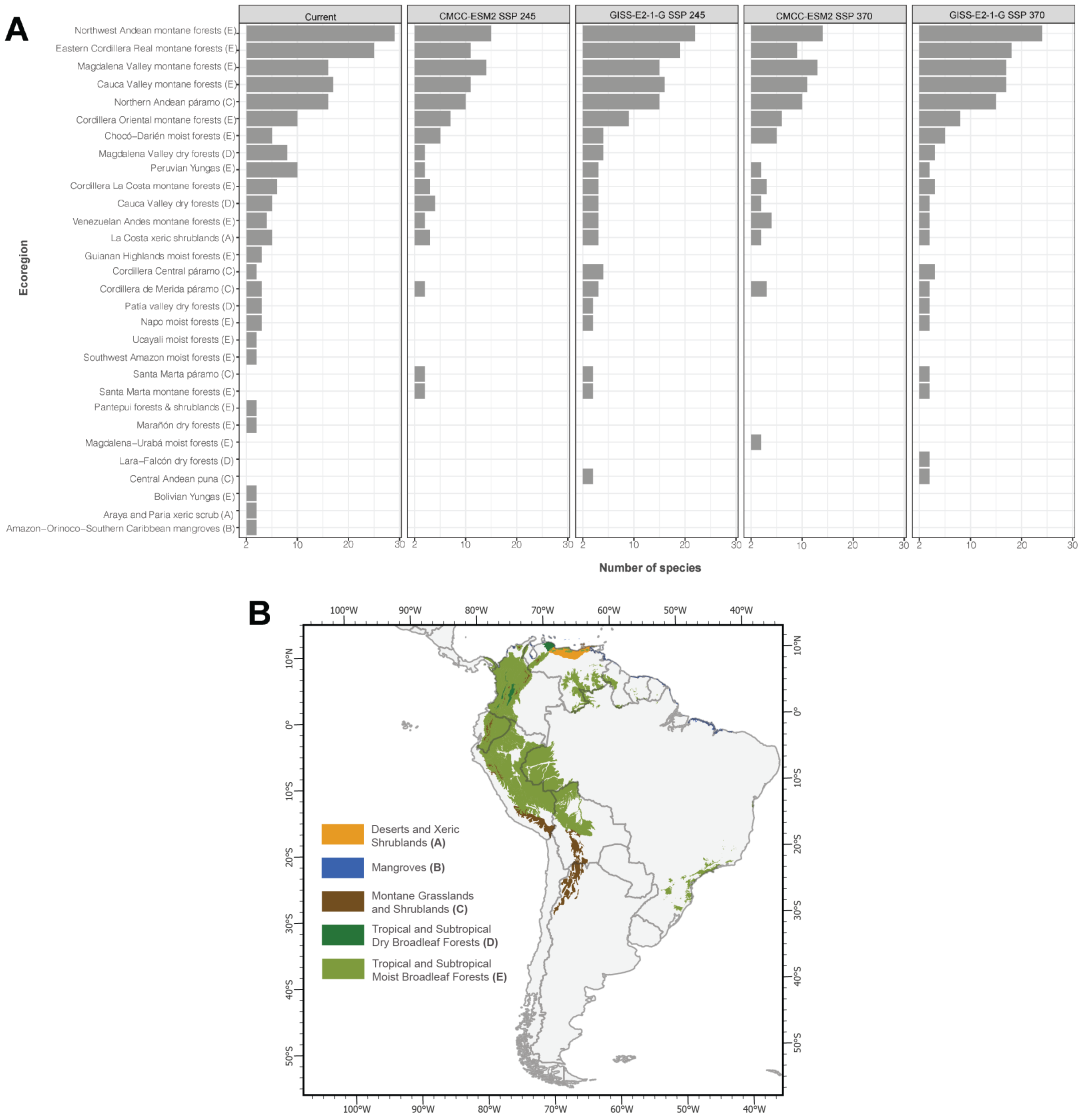
(A) Number of threatened glassfrog species by ecoregion, according to the different global climate models (GCMs) and Shared Socio-economic Pathways (SSPs). Supplementary S3 details the species present in each ecoregion, together with the corresponding number of species. (B) Distribution map of the biomes encompassing the ecoregions, highlighting the geographic areas corresponding to the glassfrog species in each ecoregion.

The current model shows that the pattern of richness of threatened species is concentrated north of the Andes and its foothills, specifically in Ecuador and Colombia (Fig. 3). This pattern is found mainly in the ecoregions Northwest Andean montane forest, Eastern Cordillera Real montane forest, Magdalena Valley montane forest, and Cauca Valley montane forest that correspond to the Tropical and Subtropical Moist Broadleaf Forests biome (Fig. 4A).

#### Scenario SSP2-4.5

The results for the CMCC-ESM2 model show a more dispersed pattern of taxonomic diversity across the Andes Mountain range and its foothills. In the eastern foothills of the range, particularly in Ecuador near Sumaco National Park, several pixels exhibit high taxonomic diversity. Similarly, in Colombia, regions with high values are observed in the Department of Cauca near Popayán and further north in the Department of Caldas. The ecoregions with the highest species richness in this model largely align with those identified in the current model, with the Northwest Andean Montane Forest emerging as the most species-rich ecoregion. However, there is a notable decline in species richness within the Eastern Cordillera Real Montane Forest ecoregion, accompanied by the extinction of some species in other ecoregions (Fig. 4A). In comparison, the GISS-E2-1-G model highlights taxonomic diversity primarily in the southeastern Andes Mountains of Colombia (between 1° and 4° latitude) and the eastern foothills of the northeastern Andes Mountains in Ecuador. The pattern of species richness across ecoregions remains consistent with previous models, again identifying the Northwest Andean Montane Forest as the ecoregion with the highest species richness.

#### Scenario SSP3-7.0

**Figure 5:**
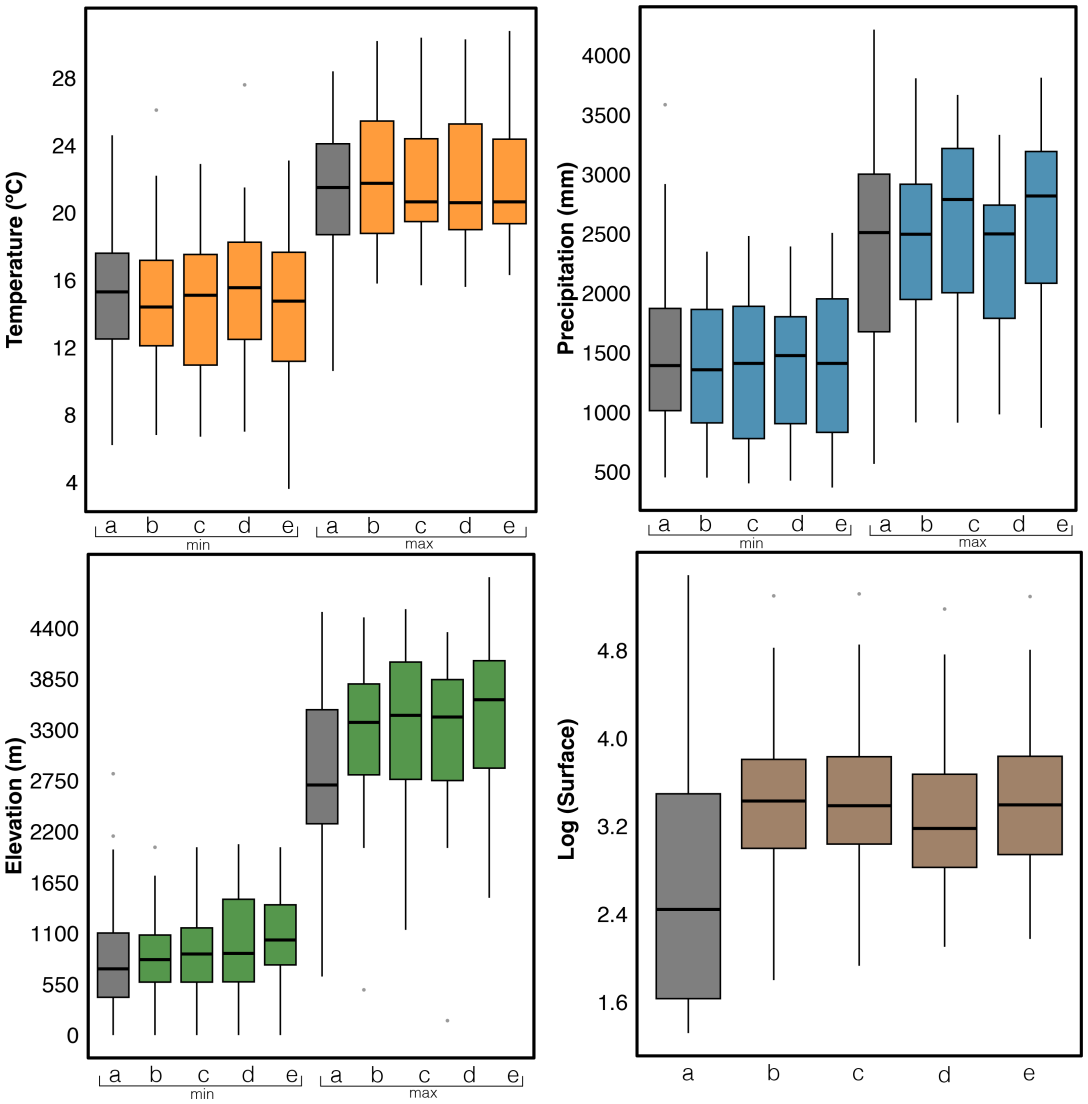
Boxplots of variables for the current and future distributions of threatened glassfrogs for each global climate models (GCMs) and Shared Socio-economic Pathways (SSPs). Temperature (min-max), Precipitation (min-max), Elevation (min-max) and Surface logarithm. Letters on the *x*-axis correspond to GCMs: a = Current, b = CMCC-ESM2 SSP2-4.5, c = GISS-E2-1-G SSP2-4.5, d = CMCC-ESM2 SSP3-7.0, and e = GISS-E2-1-G SSP3-7.0. Detailed values for each species are provided in Supplementary S4.

The results for the CMCC-ESM2 model show that the distribution pattern is like that observed in previous models, with the highest taxonomic diversity concentrated in the northern Andes and its foothills. In Ecuador, pixels with high values are identified in the eastern foothills of the mountain range, specifically in the Cayambe-Coca and Sumaco Napo-Galeras National Parks, as well as to the west of the mountain range. In Colombia, by contrast, pixels with high values are observed in the Department of Cauca, near the Puracé National Natural Park, and south of the Farallones de Cali National Natural Park. Relevant areas are also found in the center-west of the country, particularly in the departments of Caldas and Antioquia. However, this model projects a decline in habitat suitability for glassfrogs in southern regions, particularly in Peru and Bolivia. The Northwest Andean Montane Forest ecoregion continues to harbor the highest number of threatened glassfrog taxonomic diversity. Additionally, this is the only model in which species extinction is observed in the Magdalena Valley dry forests ecoregion (Fig. 4B). Finally, the GISS-E2-1-G model projects a higher taxonomic diversity in southern Colombia, particularly in the departments of Cauca and Nariño. Some of the pixels with high richness values are in protected areas such as the Munchique National Natural Parks and the Doña Juana-Cascabel Volcanic Complex. Likewise, in the eastern foothills of the Andes Mountains in Ecuador, between Cayambe-Coca and Sumaco National Parks, a pixel with high species richness has been identified. According to previous models, the ecoregion that will harbor the highest number of threatened species is the Northwest Andean Montane Forest, followed by the Eastern Cordillera Real Montane Forests ecoregion (Fig. 4B). The values for temperature, precipitation, elevation, and surface area for each species of threatened glassfrog are presented in Table S2. While the minimum and maximum values of these variables for each GCM in each SSP are provided in Fig. 5.

### Species under high risk of extinction

Our modeling results suggest that between 2061–2080, twelve threatened glassfrog species would lose suitable conditions for survival under at least one General Climate Model (GCM) and one Shared Socio-economic Pathway (SSP). Of these species, six are projected to completely lose climatic suitability under both GCMs and SSP scenarios (Table 2). These species (*Centrolene altitudinalis*, *Centrolene condor*, *Hyalinobatrachium duranti*, *Nymphargus lasgralarias*, *Nymphargus prasinus* and *Vitreorana helenae*) are distributed in northern South America, inhabiting five ecoregions, ranging in elevation from 89 to 3,680 m. In evolutionary perspective, *Vitreorana helenae* is the species with the longest evolutionary time, evolving for approximately 12 million years. It is currently categorized as Vulnerable (VU) and inhabits the Guiana Highlands Moist Forests, Guianan Savanna and Negro-Branco Moist Forests ecoregions. In contrast, *Centrolene altitudinalis* is the species with the shortest evolutionary time, with about 2.9 million years of evolution. This species is classified as Endangered (EN), and its distribution is restricted to the Mérida Mountain range in Venezuela.

**Table 2:** Species projected to lose their entire climatic identity by 2061–2080 based on the SDM, with all predictor variables representing current conditions.

**Species**	**IUCN category**	**Current distribution**	**Temperature (°C)**	**Evolution time** **(Ma)**	**Precipitation (mm)**	**Elevation (m)**	**CMCC-ESM2 SSP2-4.5**	**GISS-E2-1-G SSP2-4.5**	**CMCC-ESM2 SSP3-7.0**	**GISS-E2-1-G SSP3-7.0**
Centrolene altitudinalis^*^	EN	Venezuelan Andes montane forests	13.8–15.8	2.9	1,000–1,090	1,735–3,544	Loss of climatic suitability	Loss of climatic suitability	Loss of climatic suitability	Loss of climatic suitability
Centrolene condor^*^	EN	Eastern Cordillera Real montane forests	16.9–19.6	5.0	1,318–1,623	946–2,674	Loss of climatic suitability	Loss of climatic suitability	Loss of climatic suitability	Loss of climatic suitability
*Centrolene medemi*	EN	Cordillera Oriental montane forests Eastern Cordillera Real montane forests Magdalena Valley dry forests Napo moist forests	16.8–22.4	–	1,872–2,509	640–2,382	Loss of climatic suitability	–	Loss of climatic suitability	–
*Centrolene pipilata*	CR	Eastern Cordillera Real montane forests	16.3–18.7	3.9	2,198–3,001	1,103–2,457	Loss of climatic suitability	–	Loss of climatic suitability	–
Hyalinobatrachium duranti^*^	EN	Venezuelan Andes montane forests	13.7–17.4	6.7	946–1,129	1,453–3,680	Loss of climatic suitability	Loss of climatic suitability	Loss of climatic suitability	Loss of climatic suitability
*Nymphargus balionotus*	EN	Northwest Andean montane forests	14.3–22.2	16.4	1,564–2,706	525–2,751	Loss of climatic suitability	–	Loss of climatic suitability	–
*Nymphargus bejaranoi*	EN	Bolivian montane dry forests Bolivian Yungas Central Andean puna Southern Andean Yungas	14.1–19	9.5	652–2,262	1,080–3,596	Loss of climatic suitability	–	Loss of climatic suitability	–
*Nymphargus cariticommatus*	EN	Eastern Cordillera Real montane forests	12.1–21.3	8.2	1,082–1,676	864–3,517	Loss of climatic suitability	–	Loss of climatic suitability	–
Nymphargus lasgralarias^*^	EN	Northwest Andean montane forests	15.4–18.4	7.3	1,036–2,185	1,293–2,663	Loss of climatic suitability	Loss of climatic suitability	Loss of climatic suitability	Loss of climatic suitability
*Nymphargus megistus*	EN	Cauca Valley montane forests Northwest Andean montane forests	12–21.5	3.6	1,729–3,035	489–3,629	Loss of climatic suitability	–	Loss of climatic suitability	–
Nymphargus prasinus^*^	VU	Cauca Valley montane forests Northwest Andean montane forests	16–20	–	1,627–3,462	883–2,638	Loss of climatic suitability	Loss of climatic suitability	Loss of climatic suitability	Loss of climatic suitability
Vitreorana helenae^*^	VU	Guiana Highlands moist forests Guianan savanna Negro-Branco moist forests	21.5–25.3	12.0	1,683–2,427	89–1,401	Loss of climatic suitability	Loss of climatic suitability	Loss of climatic suitability	Loss of climatic suitability

**Notes.**

* Indicates species consistently projected to lose all suitable climatic conditions across both Global Climate Models (GCMs) and Shared Socio-economic Pathways (SSPs).

### Spatial phylogenetic diversity

Differences between current phylogenetic diversity and GCMs projections indicate a consistent future decline. Under the SSP2-4.5 scenario, CMCC-ESM2 projects an average loss of 34.2% of phylogenetic diversity, whereas GISS-E2-1-G estimates a lower reduction of 22.6%. Under SSP3-7.0 (high impact scenario), CMCC-ESM2 again predicts the highest decline (41.3%), while GISS-E2-1-G projects a loss of 24.8%. Overall, despite variations in magnitude between models, all projections consistently indicate that the phylogenetic diversity of threatened glassfrogs could decline substantially in the future.

#### Current potential scenario

**Figure 6:**
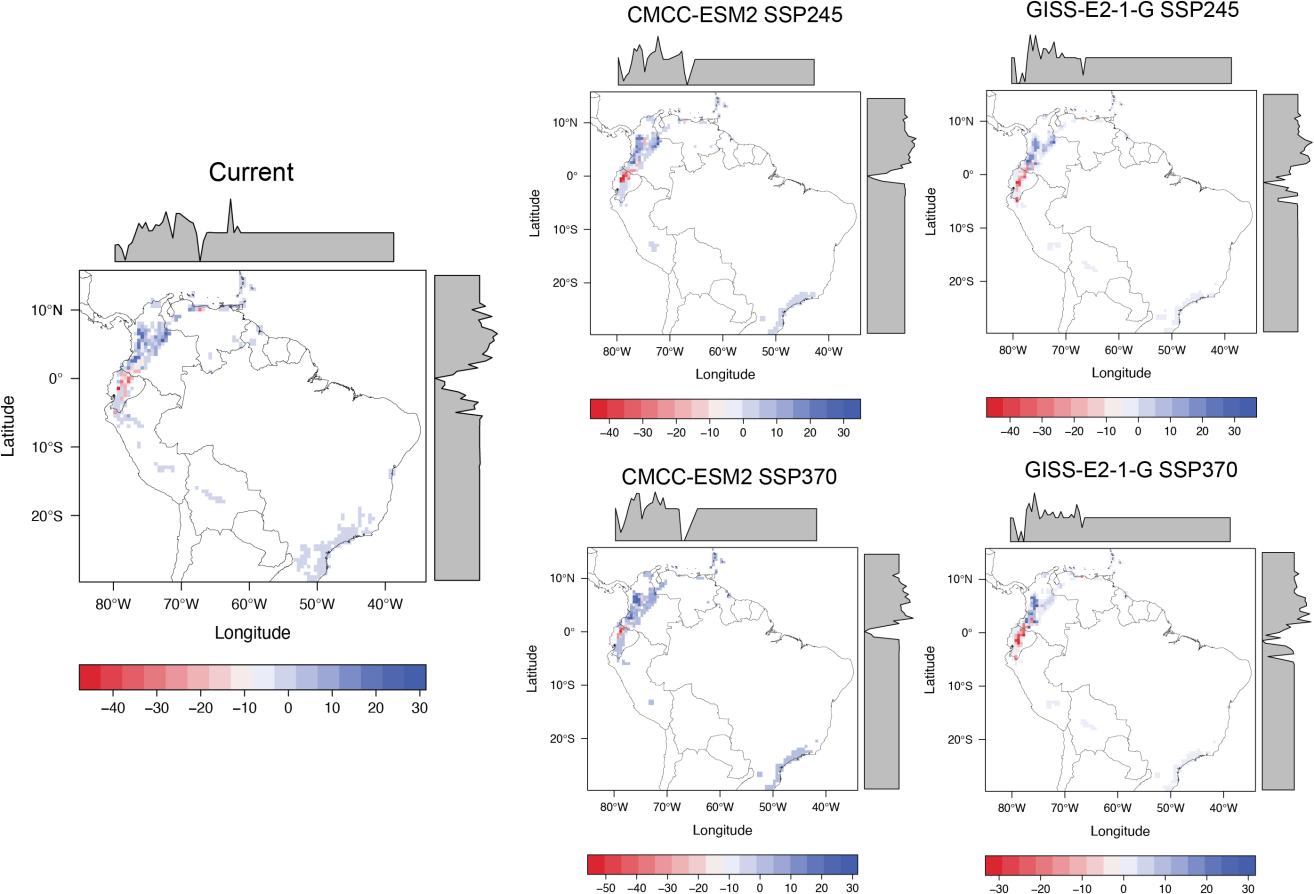
Residuals between phylogenetic diversity (PD) and taxonomic diversity (TD) of threatened Neotropical glassfrogs, where positive residuals indicate few recent speciation events and high dispersal rates, while negative residuals suggest many recent speciation events and/or low dispersal rates.

The map resulting from the residuals of the Locally Estimated Scatterplot Smoothing between PD ∼TD shows a consistent pattern in which the most recent speciation events and/or low dispersal rates (negative residuals) are mainly concentrated in northern Ecuador, specifically in the eastern (Eastern Cordillera Real montane forests) and western (Northwest Andean montane forests) foothills of the Andes. Some pixels with similar characteristics are also heterogeneously distributed in regions of Venezuela (Guianan Highlands moist forests and Guianan piedmont moist forests), Colombia (Magdalena-Urabá moist forests and Eastern Cordillera Real montane forests) and Peru (Peruvian Yungas). However, the pattern of lowest recent speciation events and/or high dispersal rates (positive residuals) of threatened glassfrogs are mostly distributed in Colombia (Northwest Andean montane forests, Northern Andean páramo, Magdalena Valley montane forests and Cauca Valley montane forests) and Venezuela (Venezuelan Andes montane forests and La Costa xeric shrublands) (Fig. 6).

#### Scenario SSP2-4.5

For the CMCC-ESM2 model the map of residuals shows that the negative residuals are mainly concentrated in northern Ecuador, specifically in the western foothills (Northwest Andean montane forests). Similarly, some pixels are identified in the eastern foothills of the Eastern Cordillera Real Montane Forests ecoregion extending from southern Ecuador to southern Colombia. Positive residuals are mostly distributed in Colombia, along the Central Cordillera and north of the Eastern Cordillera. The GISS-E2-1-G model projects a similar pattern, although more dispersed. The map of the residuals shows that most pixels with negative values are located along the Andes of Ecuador from north to south, and a few pixels in southern Colombia (Department of Nariño). While the positive residuals are observed in Colombia, and as in the previous model along the Cordillera Central and the Cordillera Oriental. Residuals tending to zero are more evident in the north of South America, especially in Venezuela and Guyana, as well as in the extreme south, in regions such as Peru, Bolivia and Brazil (Fig. 6).

#### Scenario SSP3-7.0

The map of the residuals of the first CMCC-ESM2 compared to the previous models shows that the negative residuals are very restricted to northwestern Ecuador (Pichincha province) in the Northwest Andean montane forests ecoregion. We can identify at least two pixels with similar values, one near Sumaco National Park (Ecuador) and the other, south of the Reserva Forestal Protectora de la Cuenca Alta del Río Mocoa (Colombia). The positive residuals for this model suggest that they are distributed according to the previous models (in the Cordillera Central and north of the Cordillera Oriental, Colombia), although with less intensity. Finally, the residual map of the GISS-E2-1-G shows that the negative residuals are distributed on both sides of the foothills of the Andes in Ecuador, in a wider distribution compared to the previous model. The model also identifies certain pixels in southern Colombia, in the departments of Cauca and Nariño, within the Eastern Cordillera Real montane forest ecoregion, that present negative residuals. Nevertheless, the positive residuals are mostly concentrated in the north of the Central Cordillera (Northwest Andean montane forests and Cauca Valley montane forests) in Colombia. In this model, pixels with values close to zero are even more evident throughout the future distribution of threatened glassfrogs (Fig. 6).

### Conservation

#### Distribution in protected areas

**Figure 7:**
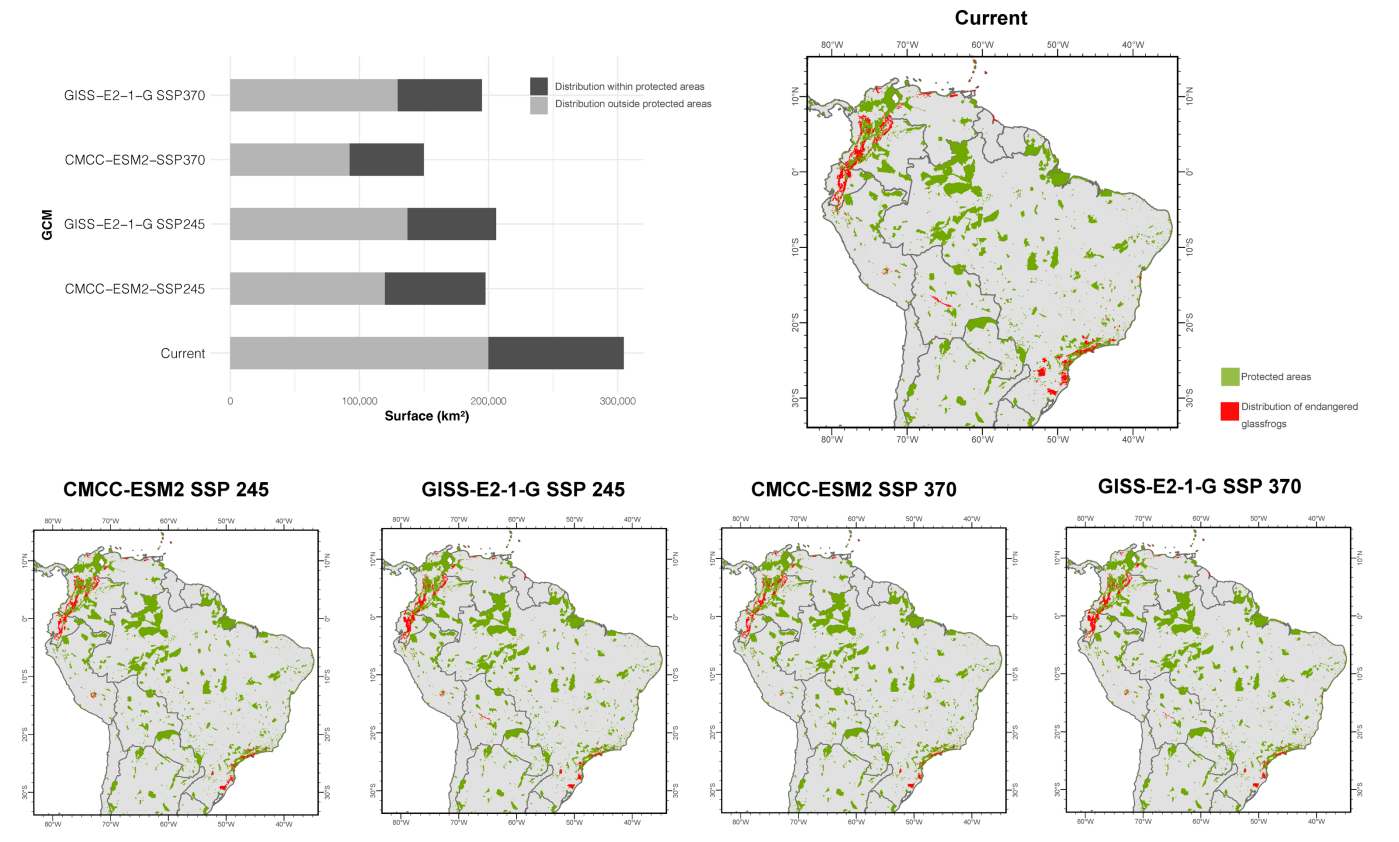
Area inside and outside protected areas for the current and future distribution of threatened glassfrogs. Protected areas were obtained from the World Database on Protected Areas (WDPA).

In the SSP2-4.5 scenario, CMCC-ESM2 projects, on average, a 40.7% contraction area of the threatened glassfrog species modeled. The estimated area of distribution is 197,507 km^2^, of which 39.4% is within protected areas (Fig. 7). Twelve species of glassfrogs will face a complete contraction (100%) in their area of distribution (Table 3). Among the most affected genera, *Vitreorana* will experience the highest contraction in its range, with an approximate reduction of 78.2%. It is followed by *Centrolene*, whose distribution area will be reduced by 66.5%. The other genera will have an area contraction of less than 55%, the only genus that shows a gain in its distribution area is *Sachatamia*. The GISS-E2-1-G projects an average area contraction of 49.3% in the distribution of the modeled species, which is an increase of 8.6% compared to the previous model. According to the estimate, the potential area of distribution covers 205,827 km^2^, of which 33.3% is within protected areas (Fig. 7). Six species will experience a complete contraction (100%) in their ranges. As in the previous model, the genus *Vitreorana* is expected to have a high contraction area of 60.5%, followed by the genus *Ikakogi* (one species, *I. tayrona*) where its distribution will decrease by 77.5%. The other genera will have an area contraction of less than 32%, the only genus that will have a gain in distribution is *Cochranella* (one species, *C. litoralis*).

**Table 3:** Percentage of area of contraction of species modeled. The table includes the Evolutionarily Distinct and Globally Endangered (EDGE) index.

**Species**	**CMCC-ESM2 SSP2-4.5**	**GISS-E2-1-G SSP2-4.5**	**CMCC-ESM2 SSP3-7.0**	**GISS-E2-1-G SSP3-7.0**	**EDGE**
*Centrolene altitudinalis*	100.0	100.0	100.0	100.0	2.69
*Centrolene ballux*	58.2	45.2	89.4	41.4	2.38
*Centrolene buckleyi*	28.0	23.0	37.4	30.9	2.61
*Centrolene condor*	100.0	100.0	100.0	100.0	3.64
*Centrolene geckoidea*	55.3	42.7	54.9	41.4	4.09
*Centrolene heloderma*	65.3	46.2	72.8	61.9	2.72
*Centrolene huilensis*	−25.8	76.3	74.2	65.6	2.51
*Centrolene lynchi*	72.7	58.5	85.2	68.8	2.29
*Centrolene medemi*	100.0	16.2	100.0	−38.7	–
*Centrolene pipilata*	100.0	−96.1	100.0	−123.0	3.29
*Centrolene quindianum*	70.7	−2.2	78.9	1.5	–
*Centrolene sanchezi*	89.2	−74.3	75.7	−64.8	3.06
*Centrolene solitaria*	51.7	87.4	93.1	55.2	–
*Cochranella litoralis*	27.8	−248.9	15.6	−256.7	3.47
*Hyalinobatrachium duranti*	100.0	100.0	100.0	100.0	3.64
*Hyalinobatrachium esmeralda*	20.3	−29.2	33.3	−25.1	3.14
*Hyalinobatrachium guairarepanense*	45.2	60.7	52.4	81.0	3.17
*Hyalinobatrachium orientale*	54.1	43.3	59.6	41.4	3.04
*Ikakogi tayrona*	48.0	77.6	42.4	80.0	3.71
*Nymphargus anomalus*	90.4	49.3	83.3	66.0	2.82
*Nymphargus balionotus*	100.0	84.6	100.0	89.2	3.43
*Nymphargus bejaranoi*	100.0	56.7	100.0	74.6	3.21
*Nymphargus buenaventura*	29.4	−21.6	21.5	19.6	3.03
*Nymphargus cariticommatus*	100.0	24.5	100.0	21.0	2.80
*Nymphargus garciae*	9.0	13.3	53.7	−67.6	3.07
*Nymphargus lasgralarias*	100.0	100.0	100.0	100.0	3.03
*Nymphargus megistus*	100.0	91.1	100.0	75.6	3.52
*Nymphargus pluvialis*	−87.0	−17.4	46.4	14.5	3.21
*Nymphargus prasinus*	100.0	100.0	100.0	100.0	–
*Nymphargus rosada*	49.9	45.5	88.6	74.2	2.80
*Nymphargus ruizi*	7.4	−8.1	77.2	−20.4	–
*Nymphargus siren*	21.2	8.4	24.5	3.9	2.88
*Rulyrana adiazeta*	3.3	22.1	13.1	36.9	3.08
*Sachatamia electrops*	−50.0	59.6	53.4	79.4	3.42
*Sachatamia punctulata*	−104.5	4.7	−35.0	−3.3	3.03
*Vitreorana antisthenesi*	94.3	71.7	88.6	41.4	3.07
*Vitreorana helenae*	100.0	100.0	100.0	100.0	3.45
*Vitreorana parvula*	40.3	49.4	53.9	54.6	3.07

For the SSP3-7.0 scenario, the CMCC-ESM2 model estimates an average area contraction of 53.8% for the species modeled. The total projected area occupied by the species in the future, according to this model, is 149,892 km^2^, of which 38.4% is within protected areas (Fig. 7). Twelve species will have a complete contraction (100%) in their ranges (Table 3). Coinciding with previous models, the genus *Vitreorana* presents a high percentage of contracted area (80.8%). However, this model projects that the genus *Centrolene* will have the highest area of contraction in its range, 81.6%. The other genera show significant percentages of area contraction but below the 76% threshold. The GISS-E2-1-G projects, on average, a 54.5% contraction in the area of distribution of the modeled species. The total area estimated for this projection is 194,701 km^2^, of which 33.4% is within protected areas. Six species will face a complete contraction (100%) in their ranges. In this model, the genus *Ikakogi* (one species, *I. tayrona*) will face the highest area contraction (79.9%), followed by the genus *Vitreorana* with 65.3%. The other genera will have an area contraction below 50%. Only *Cochranella* (one species, *C. litoralis*) will have a gain in distribution.

### Evolutionarily Distinct and Globally Endangered

**Figure 8:**
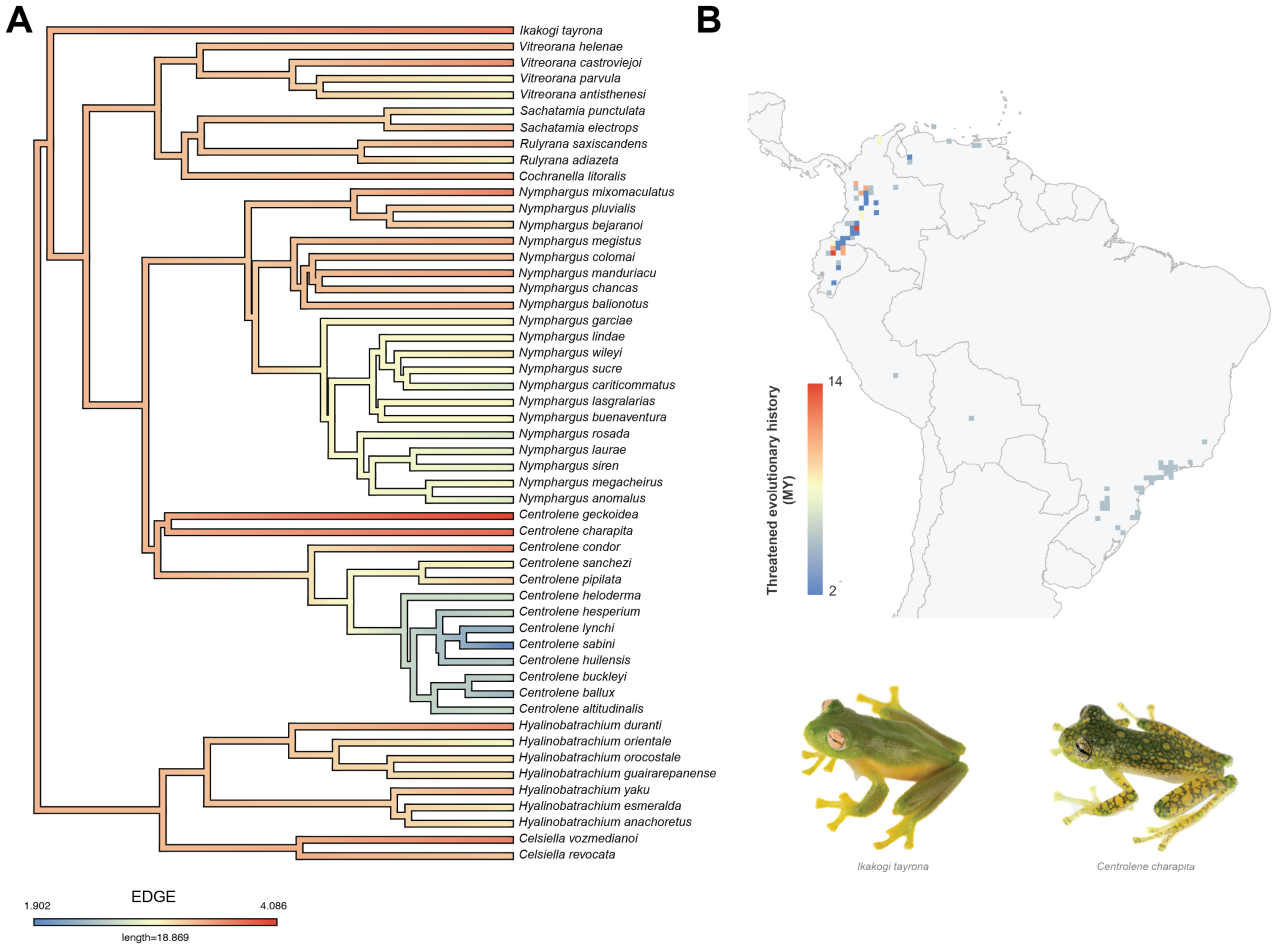
(A) Phylogeny of threatened glassfrogs based on Evolutionary Distinctiveness and Global Endangerment (EDGE). (B) Distribution of threatened glassfrogs evolutionary history.

The species with the highest EDGE value (4.09) is *Centrolene geckoidea*, which inhabits the northern Andes, between Ecuador (historical distribution) and Colombia. This value indicates that it is the species with the highest priority of conservation due to its evolutionary uniqueness and critical risk of extinction. It is followed by *Centrolene charapita* (3.86), which is restricted to southern Ecuador and northern Peru. *Nymphargus mixomaculatus* (3.72) comes next, with a distribution restricted to the province of Huánuco, Peru. The other species have values less than or equal to 3.71 (Fig. 8A; Table 2). The species with the lowest EDGE value (1.9) is *Centrolene sabini*, which is recorded only in Paucartambo province, Peru. This value suggests that, despite being classified as Vulnerable (VU), it has a low evolutionary distinction.

The distribution pattern of threatened glassfrog species according to the Evolutionarily Distinct and Globally Endangered (EDGE) index shows that the pixels with the highest values (∼14 My) are located in northern South America (Ecuador and Colombia), in the ecoregion of Northwest Andean montane forests (Fig. 8B). In Ecuador, they occur along the eastern and western foothills of the northern Andes, particularly in the provinces of Pichincha and Napo. In Colombia, they are found in the department of Huila, near the Puracé National Natural Park, and in the north, in the central mountain range, in the departments of Caldas and Antioquia. These locations indicate that evolutionarily unique and highly threatened species inhabit these areas. The other pixels have lower EDGE values and are distributed over most of South America, from Venezuela to Brazil (Fig. 8B).

## Discussion

### Species distribution models

One of the main limitations in the application of distribution models is the lack of species occurrence data, which can lead to a reduction in model accuracy and higher uncertainty in predictions, depending on the modeling algorithms (Wisz et al., 2008). Specifically in the case of the threatened glassfrogs, only 55% of the 69 species were modeled because the remaining species had very few occurrence records. This also enabled us to identify information gaps in Neotropics, where threatened glassfrog species are distributed, reflecting the Wallacean shortfall, that is, limited knowledge of species geographic distributions. For example, most species with few records occur in Venezuela, Peru, and Bolivia, countries that also have limited biodiversity databases and scarce open access information. This emphasizes the need to implement efforts to generate and obtain open access and high-quality data in these countries (Canhos et al., 2015; Bermudez, Pérez Mesa & Ottogalli, 2022). Besides, SDMs predict the potential distribution of a species based on its climatic niche (Naimi & Araújo, 2016). These predictions alone are very useful, as they allow us to elucidate and provide a panorama with more evidence on species distributions. However, it is also important to consider models such as deforestation, threats and changes in land use (Chowdhury, 2006; Eglington & Pearce-Higgins, 2012), especially in regions such as the Andes and the Amazon, where habitats face multiple pressures (Sierra et al., 2022; Albert et al., 2023). The integration of several models (mentioned above) with climate niche models allows the generation of more complete and useful future scenarios for conservation.

### Species extinction

Differences between GCMs and SSPs have led to some variations in projections of the future distribution of threatened glassfrogs in the Neotropics (previous section). One of these differences relates specifically to projections of absolute loss of climatic niche conditions for some species, *i.e.,* their extinction. This phenomenon is based on the niche contraction hypothesis (Scheele et al., 2017), which suggests that as a species niche contract, it becomes increasingly difficult for it to survive and adapt to the new conditions. In extreme cases, if it is unable to recolonise other areas or adapt quickly to changes, this process can lead to its extinction (Scheele et al., 2017). In fact, the projections of our models are so radical that they show that in the future (2061–2080), species that will go extinct will simply not have a chance to recolonise other areas or adapt quickly to changing climatic conditions in their niche. This is because the effects of climate variability will be drastic, especially in the Andes and the Amazon River basin (Menéndez-Guerrero, Davies & Green, 2020). It is important to mention that our models agree that at least six species will lose their climatic niche in both GCMs and SPPs for the period (2061–2080). These species have a restricted distribution and are endemic to Venezuela, Colombia and Ecuador, and are mainly associated with mountain systems (*i.e.,* Cordillera de Mérida, Cordillera Occidental, Guiana Shield and Cordillera del Cóndor). Also, this process may ultimately be constrained by climatic niche conservatism, as species with limited capacity to evolve their climatic tolerances must rely on niche tracking. If dispersal is insufficient, species may face local or global extinction due to their inability to reach suitable habitats in time (Hutter, Guayasamin & Wiens, 2013). Moreover, natural and anthropogenic barriers, such as habitat fragmentation, rivers, or unsuitable matrix habitats, can further restrict movement, potentially altering predictions of species survival under climate change. Dispersal limitations can constrain upward elevational shifts and highlight the importance of considering both species mobility and landscape connectivity in future projections (Robertson, Lips & Heist, 2008). On the other hand, the modelled species that will survive tend to increase their elevational range in search of areas with more favorable climatic conditions for their survival, which is consistent with previous studies (Forero-Medina, Joppa & Pimm, 2011; Tiberti, Mangiacotti & Bennati, 2021; Souza et al., 2023).

### Conservation implications

The results obtained have enabled the identification of both current and future areas (climatic refugia) that are of fundamental importance and priority for the conservation of threatened glassfrogs, as well as for biodiversity in general, considering that amphibians are widely recognized as model organisms for environmental change studies (Hopkins, 2007). This identification of conservation areas is supported by a robust approach, as the taxonomic diversity and, complementarily, the phylogenetic diversity of threatened glassfrog species have been assessed for current and future scenarios. It should also be considered that the spatial resolution used may not capture very small-scale refuges or fine local variations in EDGE areas, but it remains adequate for regional-scale analyses. At this scale, phylogenetic diversity and the EDGE index effectively identify key species for urgent conservation action due to their evolutionary importance. In fact, there are certain areas with high EDGE scores, which coincide with the EDGE zones of Pipins et al. (2024), where patterns of high distinctiveness and extinction risk are observed mainly in amphibians. Another important point is that complete data on the sequence of Centrolenidae species is not available. Generation of additional sequences for these species is essential to improve future analyses and enhance conservation planning.

Nevertheless, these areas, which have been identified as key to the survival of glassfrogs, need to be placed under a conservation scheme (Le Saout et al., 2013). Our results suggest that in future scenarios, approximately 36% of the distribution of threatened species will be within protected areas. Increasing this proportion is expected to be challenging due to existing threats such as climate change, habitat loss, and fragmentation driven by land-use changes (Ochoa-Ochoa & Velasco, 2024; Guayasamin, Franco-Mena & Vega-Yánez, 2025).

## Conclusions

Most endangered glassfrog species are distributed in the Andes, from Peru to Colombia. We found a gap in the information on the occurrence records of glassfrogs, mainly in Venezuela, Peru and Bolivia, which paradoxically harbor endemic species at high risk of extinction. Our current and future distribution models suggest that the northern Andes of Ecuador and Colombia, especially the Northwest Andean Montane Forests ecoregion, will be an important refuge for the taxonomic and phylogenetic diversity of these species, despite the drastic climatic changes that part of the Andes and the Amazon basin are expected to face. In addition, projections indicate that six species will entirely lose their climatic suitability under both General Circulation Models and the two Shared Socio-economic Pathways for the period 2061–2080; *Centrolene altitudinalis*, *Centrolene condor*, *Hyalinobatrachium duranti*, *Nymphargus lasgralarias*, *Nymphargus prasinus* and *Vitreorana helenae.* Furthermore, under the SSP2-4.5 scenario, an average loss of 28.4% of the phylogenetic diversity among threatened glassfrogs is projected, whereas under the SSP3-7.0 scenario, the average loss could increase to 33%. From an evolutionary and conservation perspective, based on the Evolutionarily Distinct and Globally Endangered (EDGE) index, *Centrolene geckoidea* and *Centrolene charapita* are keystone species for conservation due to their evolutionary history. This means that the loss of these lineages would be irreplaceable, as they have no close relatives and have evolved in isolation for millions of years. In contrast, the other glassfrog species modelled tend to increase their elevation range but show a decrease in their distribution area. Less than 36% of their projected range is within protected areas; since the survival of species is linked with elevational migrations, the expansion of protected areas, considering elevation gradients and corridors, is key.

##  Supplemental Information

10.7717/peerj.21165/supp-1 Supplemental Information 1 Sources of glassfrog occurrence records used for analysis

10.7717/peerj.21165/supp-2 Supplemental Information 2 Taxonomic information, voucher number, locality, and genes (12S, 16S, and ND1) of the glass frog sequences obtained from GenBank and used in this study

10.7717/peerj.21165/supp-3 Supplemental Information 3 Number of glassfrog species per ecoregion estimated for the current scenario and for four future climate scenarios (SSP245: CMCC-ESM2; SSP245: GISS-E2-1-G; SSP370: CMCC-ESM2; SSP370: GISS-E2-1-G)

10.7717/peerj.21165/supp-4 Supplemental Information 4 Minimum–maximum ranges of temperature, precipitation, elevation, and surface area logarithm for glassfrog distribution areas under the current scenario and future climate scenarios

## Additional Information and Declarations

### Data Availability

The following information was supplied regarding data availability: The raw data is available in the Supplemental Files. The data and code are available at GitHub and Zenodo: – https://github.com/mateovegayanez/-Key-areas-for-threatened-Neotropical-glassfrogs.git
– Mateo A. Vega-Yánez. (2026). mateovegayanez/-Key-areas-for-threatened-Neotropical-glassfrogs: Key areas for threatened Neotropical glassfrogs (v.01). Zenodo. https://doi.org/10.5281/zenodo.18803704.
